# Celecoxib/Cyclodextrin Eye Drop Microsuspensions: Evaluation of In Vitro Cytotoxicity and Anti-VEGF Efficacy for Retinal Diseases

**DOI:** 10.3390/pharmaceutics15122689

**Published:** 2023-11-28

**Authors:** Phatsawee Jansook, Hay Man Saung Hnin Soe, Rathapon Asasutjarit, Theingi Tun, Hay Marn Hnin, Phyo Darli Maw, Tanapong Watchararot, Thorsteinn Loftsson

**Affiliations:** 1 Faculty of Pharmaceutical Sciences, Chulalongkorn University, Bangkok 10330, Thailand; haymansaunghninsoe@gmail.com (H.M.S.H.S.); theingitun.tgt1793@gmail.com (T.T.); haymarn793@gmail.com (H.M.H.); phyodarlimaw1994@gmail.com (P.D.M.); tanapongbiggie@hotmail.com (T.W.); 2 Cyclodextrin Application and Nanotechnology-Based Delivery Systems Research Unit, Chulalongkorn University, Bangkok 10330, Thailand; 3 Thammasat University Research Unit in Drug, Health Product Development and Application (DHP-DA), Department of Pharmaceutical Sciences, Faculty of Pharmacy, Thammasat University, Pathum Thani 12120, Thailand; rathapon@tu.ac.th; 4 Faculty of Pharmaceutical Sciences, University of Iceland, Hofsvallagata 53, IS-107 Reykjavik, Iceland; thorstlo@hi.is

**Keywords:** cyclodextrin, celecoxib, eye drop, microsuspensions, cytotoxicity, anti-VEGF

## Abstract

Celecoxib (CCB), a cyclooxygenase-2 inhibitor, is capable of reducing oxidative stress and vascular endothelial growth factor (VEGF) expression in retinal cells and has been shown to be effective in the treatment of diabetic retinopathy and age-related macular degeneration. However, the ocular bioavailability of CCB is hampered due to its very low aqueous solubility. In a previous study, we developed 0.5% (*w*/*v*) aqueous CCB eye drop microsuspensions (MS) containing randomly methylated β-cyclodextrin (RMβCD) or γ-cyclodextrin (γCD) and hyaluronic acid (HA) as ternary CCB/CD/HA nanoaggregates. Both formulations exhibited good physicochemical properties. Therefore, we further investigated their cytotoxicity and efficacy in a human retina cell line in this study. At a CCB concentration of 1000 μg/mL, both CCB/RMβCD and CCB/γCD eye drop MS showed low hemolysis activity (11.1 ± 0.3% or 4.9 ± 0.2%, respectively). They revealed no signs of causing irritation and were nontoxic to retinal pigment epithelial cells. Moreover, the CCB eye drop MS exhibited significant anti-VEGF activity by reducing VEGF mRNA and protein levels compared to CCB suspended in phosphate buffer saline. The ex vivo transscleral diffusion demonstrated that a high quantity of CCB (112.47 ± 37.27 μg/mL) from CCB/γCD eye drop MS was deposited in the porcine sclera. Our new findings suggest that CCB/CD eye drop MS could be safely delivered to the ocular tissues and demonstrate promising eye drop formulations for retinal disease treatment.

## 1. Introduction

Age-related macular degeneration (AMD) and diabetic retinopathy (DR) are emerging as global health issues that are leading causes of irreversible blindness and visual impairment [1,2]. The prevalence of both conditions is expected to rise over time and approximately 288 million people will be affected with AMD by 2040 and 191 million people with DR by 2030 [2,3]. Indeed, vascular endothelial growth factor (VEGF) is a common factor involved in the pathophysiology of both DR and AMD [4]. Excessive VEGF expression in the retina causes vascular leakage and choroidal neovascularization, which mean the formation of new, abnormal blood vessels in the choroid and subretinal region [5,6], ultimately causing a breakdown of the blood–ocular barrier and allowing the influx of fluids and macromolecules from the blood into the retina, resulting in central vision impairment [7]. Laser photocoagulation and vitrectomy have been the gold standard therapy for decades. Additionally, the systematic use of anti-inflammatory, antiangiogenesis, and antihypertensive agents, antioxidants, and hypoglycemic agents has been reported as beneficial in managing AMD and DR [8,9,10]. In recent years, researchers have increasingly focused on strategies targeting VEGF [11].

Celecoxib (CCB), a selective cyclooxygenase-2 inhibitor, has shown anti-inflammatory, anti-VEGF, and antiproliferative effects on the retina cells [4]. CCB reduces VEGF secretion from retinal pigment epithelium (RPE) and exhibits antiproliferative effects on RPE and the choroid endothelium. These unique properties make CCB a potential drug candidate for the treatment of AMD and DR [4]. Recently, oral administration of CCB has been proven to reduce vascular leakage and retinal VEGF mRNA expression in diabetic rat models. However, achieving therapeutic levels in the eyes requires a very high oral dose (50 mg/kg twice daily), leading to systemic side effects and toxicity [12]. Although invasive intravitreal injections can provide effective drug levels in the targeted retina, this approach is often complicated by endophthalmitis and retinal detachment, particularly after multiple injections [13]. Likewise, intraocular and periocular injections also carry a high risk of tissue damage and ocular infections [14].

To address these challenges, in a previous study, we developed noninvasive eye drop microsuspensions (MS) containing CCB and cyclodextrin (CD) [15]. Our findings revealed that randomly methylated β-cyclodextrin (RMβCD) or γ-cyclodextrin (γCD) had a strong complex forming affinity to CCB, and the addition of a biocompatible polymer enhanced CD solubilization through ternary complex formation [15]. The developed aqueous 0.5% (*w*/*v*) CCB eye drop MS, containing RMβCD or γCD and hyaluronic acid (HA), exhibited favorable physicochemical properties, including pH, osmolality, and viscosity, along with good mucoadhesion. Moreover, they exhibited a high permeation flux across various tested membranes [16]. In the present study, we conducted further investigations into the safety of our developed CCB/CD eye drop MS using in vitro hemolysis, the hen’s egg test-chorioallantoic membrane, cell viability, and short-time exposure tests. Additionally, the anti-VEGF effects (both protein and mRNA levels) in human retinal cell line were assessed using a western blot assay and a real-time polymerase chain reaction assay to evaluate the efficacy of the drug. Finally, we investigated the ex vivo transscleral diffusion of CCB/CD eye drop MS through porcine sclera.

## 2. Materials and Methods

### 2.1. Materials

Celecoxib (CCB) was kindly donated by Unison Laboratories Co., Ltd., (Bangkok, Thailand). Randomly methylated β-cyclodextrin (RMβCD) with molar substitution of 1.8 (MW 1312 Da) was purchased from Wacker Chemie (Munich, Germany) and γ-cyclodextrin (γCD) was kindly gifted by Ashland (Wilmington, DE, USA). Hyaluronic acid (HA), MW 1–1.4 MDa was from Soliance (Pomacle, France); ethylenediaminetetraacetic acid disodium salt dihydrate (EDTA) and sodium chloride (NaCl) were from Ajax Finechem Pty Ltd. (Taren Point, Australia); and benzalkonium chloride (BAC) was from Sigma–Aldrich (St. Louis, MO, USA). All other chemicals used were of analytical reagent grade purity. Milli-Q (Millipore, Billerica, MA, USA) water was used for the preparation of all solutions. The Statens Seruminstitut Rabbit Cornea (SIRC) and human retinal pigment epithelial (ARPE-19) cell lines were purchased from American Type Culture Collection (ATCC) (Manassas, VA, USA). All reagents used in cell culture were purchased from Invitrogen (Thermo Fisher Scientific, Waltham, MA, USA).

### 2.2. Preparation and Characterizations of CCB Eye Drop Microsuspensions (MS)

Previously, preparation of the CCB/RMβCD MS and CCB/γCD MS eye drops and their characterization has been described [16]. A 0.5% (*w*/*v*) solution of CCB was suspended in an aqueous CD solution (7.5% *w*/*v* RMβCD or 10% *w*/*v* γCD). Then, 0.1% (*w*/*v*) EDTA and 0.02% (*w*/*v*) BAC were added to the suspension. A mixer mill (RETSCH^®^ MM400, Haan, Germany) with 2 mm zirconium beads was used to reduce the size of the aqueous CCB eye drop suspensions and the process was performed at 25 Hz for 30 min. Subsequently, 0.5% (*w*/*v*) HA was added and mixed until it was completely dissolved. The pH of the resulting CCB eye drop MS was adjusted to 7.4 with sodium hydroxide (NaOH) and tonicity was adjusted with NaCl. The suspension was further sonicated in an ultrasonic bath (GT Sonic, China) at 70 °C for 1 h, and finally, water for injection was added to obtain the desired volume. CCB ophthalmic MS containing RMβCD or γCD were designated as CCB/RMβCD MS and CCB/γCD MS, respectively. These CCB eye drop MS contain micro- and nanoparticles of free CCB, solid CCB/CD complexes, and dissolved CCB/CD nanoaggregates. The particle size of CCB/RMβCD MS and CCB/γCD MS is less than 8 μm, which has been previously reported as acceptable and nonirritating to the eye [16].

The appearance of CCB eye drop MS was visually inspected, and the pH value was measured using a pH meter (METTLER TOLEDO™, SevenCompact S220-Basic, GmbH, Giessen, Germany) at room temperature. The osmolality was measured in an osmometer (OSMOMAT 3000 basic, Genotec GmbH, Berlin, Germany) using the freezing point depression principle at room temperature. A viscometer (Sine-wave Vibro SV-10, A&D Company, Tokyo, Japan) was used to determine the viscosity of each formulation. The surface tension of the formulation was measured by using a dynamic contact angle meter and tensiometer (DCAT 21, Dataphysics instrument GmbH, Filderstadt, Germany) via a Wilhelmy plate. The re-dispersion time, i.e., the time required to obtain a uniform suspension, was determined after the vial was placed in an upright position and motionless for 5 days. A mechanical shaker (Stuart Scientific, Nottingham, UK) was used to roll the container in a horizontal position at 75 rpm and time for the achievement of the homogenously suspended formulation was recorded. Total CCB content and dissolved content were analyzed using reversed-phase high-performance liquid chromatography (HPLC), as detailed in our previous report [16]. Each measurement was conducted in triplicate, and the results are expressed as the mean values ± standard deviation (SD).

### 2.3. Hemolysis Activity

Sheep blood was supplied by the Faculty of Veterinary Science, Chulalongkorn University. Initially, the blood sample was centrifuged at 4000 rpm for 20 min. The supernatant and the buffy coat were discarded via pipetting. The erythrocytes (RBCs) were washed three times with phosphate buffer saline (PBS, pH 7.4) and then resuspended. It was noted that the hematocrit value of RBCs in PBS was 40%. After appropriate dilution, RBCs were counted using a hemocytometer (Boeco, Hamburg, Germany). Following this, CCB eye drop MS was added to the suspended RBCs and diluted with PBS to achieve a final CCB concentration range of 25–1000 μg/mL. The samples were agitated in a shaking incubator at 37 °C and 100 rpm for 30 min and then transferred in an ice bath to stop hemolysis. Finally, the samples were centrifuged at 3000 rpm for 5 min, and the supernatant was analyzed for free hemoglobin concentration at 576 nm using a UV–VIS spectrophotometer (Model UV-1601, Shimadzu, Tokyo, Japan) [17]. The percentage of hemolyzed RBC (% hemolysis) was determined using the following equation.

(1) $$\%\;\mathrm{Hemolysis}\;=\frac{\left(\mathrm{Abs}-{\mathrm{Abs}}_{0}\right)}{\left({\mathrm{Abs}}_{100}-{\mathrm{Abs}}_{0}\right)}\;\times \;100$$

where Abs, Abs_0_, and Abs_100_ were the absorbances for the sample, control with PBS, and control with distilled water, respectively.

### 2.4. Irritation Study by Hen’s Egg Test-Chorioallantoic Membrane (HET-CAM)

The cytocompatibility of CCB/RMβCD MS and CCB/γCD MS were determined using the HET-CAM assay [18]. Firstly, fertile broiler chicken eggs were hatched at 38.0 ± 0.5 °C with a relative humidity of 58.0 ± 2.0% for 9 days in an automatic rotation incubator. The rotation of the incubator was stopped on day 8 to position the air sac in the wider part of the egg for an additional day. On day 9, the outer eggshell was carefully removed, and then 300 µL of CCB eye drop MS was directly applied onto the chorioallantoic membrane. The irritation potential was observed at fixed time intervals of 0.5, 2, and 5 min. In this study, 0.1% (*w*/*v*) NaOH solution served as a positive control (C+), and 0.9% (*w*/*v*) NaCl solution served as a negative control (C−). The irritation scores (IS) ranging from 0 to 21, were recorded according to Luepke (1985) [19], and irritation was classified as follows: (I) hemorrhage (vessels bleeding), (II) vascular lysis (disintegration of blood vessel) and (III) coagulation (intra- and/or extravascular denaturation of protein). The experiment was performed in triplicate.

### 2.5. In Vitro Cytotoxicity Study

#### 2.5.1. Short-Time Exposure (STE)

SIRC cells were used for performing the STE test to determine the cytotoxic effect of chemicals on corneal epithelium damage and eye irritation potential [20]. Briefly, SIRC cells (ATCC, Manassas, VA, USA) were grown in a complete medium and incubated at 37 °C in a 5% carbon dioxide (CO_2_) humidified air incubator. The complete medium used in this study consisted of Eagle’s Minimum Essential Medium (EMEM; ATCC, Manassas, VA, USA), 10% fetal bovine serum (FBS), and 1% penicillin/streptomycin solution. The cells were seeded in 96-well plates at a density of 1 × 10^5^ cells/well/100 µL and allowed to grow at 37 °C for 24 h. Subsequently, the cells were treated with 200 µL of formulations, i.e., CCB/RMβCD MS and CCB/γCD MS and their respective blanks, at concentrations of 5% and 0.05% in normal saline. The blanks of each formulation consist of excipients, except for the CCB, and follow the procedure of preparation as described in Section 2.2. Blanks of CCB/RMβCD MS and CCB/γCD MS were designated as blank RMβCD MS and blank γCD MS, respectively. The coefficient of variation (%CV) of SIRC cells was evaluated after a 5 min exposure, and ocular irritation was graded using scores of 1, 2, and 3 to indicate minimal, moderate, and severe conditions, respectively. Then, the total eye irritation score was calculated by summing the scores obtained from both 5% and 0.05% of each test [20].

#### 2.5.2. Cell Viability (CV) Test

The methyl thiazolyl-diphenyl-tetrazolium bromide (MTT) assay was employed to further investigate in vitro cytotoxicity [21,22] and to provide information for determining drug efficacy. CCB/RMβCD MS, CCB/γCD MS, and their respective blanks were tested to determine the toxicity in the ARPE-19 cell line (ATCC, Manassas, VA, USA). ARPE-19 cells express angiogenic factor, i.e., VEGF in retinal diseases [23]. In brief, the cells were grown in Dulbecco’s modified Eagle’s medium F12 (DMEM/F12; ATCC, Manassas, VA, USA), supplemented with 10% FBS and 1% penicillin/streptomycin solution, and maintained at 37 °C in a 5% CO_2_ humidified air incubator. Cells were seeded in 96-well plates at a density of 1 × 10^4^ cells/well/100 µL. Next, cells were treated by adding 100 µL of the test sample at concentrations ranging from 12.5 to 500 µg/mL to each well. After 24 h of incubation, cells were rinsed twice with PBS (pH 7.4). Subsequently, MTT solution in PBS (pH 7.4) was added to each well and incubated for 4 h. Formazan crystals were dissolved in isopropanol (100 µL/well) with 0.04 M HCl. The optical density (OD) in each well was measured at 570 nm using Fluostar Omega microplate reader (BMG Labtech, Ortenberg, Germany). The %CV was then computed according to Equation (2). A test sample was considered lethal to the cells when the %CV was less than 70%.

(2) $$\mathrm{CV}\;\left(\%\right)=\frac{{\mathrm{OD}}_{\mathrm{sample}}}{{\mathrm{OD}}_{\mathrm{control}}}\;\times 100$$

where OD_sample_ means the OD of media in each well that contains the cells treated with the drug sample, and MTT solution, and OD_control_ means the OD media without drug samples.

### 2.6. Anti-VEGF Activity

#### 2.6.1. Hypoxia Exposure

Cobalt chloride (CoCl_2_) was employed to simulate a hypoxic state by inducing the activity of hypoxia-inducible factor 1-alpha (HIF-1α), leading to the release of VEGF-A in retinal epithelial cells [24]. Our preliminary data demonstrated that a concentration of 100 µM CoCl_2_ was not toxic to ARPE-19 cells. Prior to CoCl_2_ exposure, the cells were cultured to establish a confluent monolayer in a complete medium. Subsequently, the culture medium was replaced with fresh medium containing 100 µM CoCl_2_. After a 24 h induction period, the medium containing CoCl_2_ was removed, and the cells were further incubated with samples (i.e., CCB in PBS, CCB/RMβCD MS, CCB/γCD MS, and their respective blanks). The well without treatment was replaced by the medium and designated as a negative control.

#### 2.6.2. Western Blot

A western blot assay was carried out to assess the anti-VEGF activity at the protein level [25]. After 24 h treatment with samples, ARPE-19 cells were washed with PBS and lysed. The protein was extracted by adding radioimmunoprecipitation assay buffer (10×, cat no. 9806S; Cell Signaling Technology, Danvers, MA, USA), supplemented with a protease inhibitor cocktail (100×, cat. no. 5871; Cell Signaling Technology, Danvers, MA, USA). The lysed cells were then centrifuged at 4 °C, 12,000 rpm for 5 min, and the supernatant was collected. The protein concentration was determined using a microplate reader (Fluostar Omega, BMG Labtech, Ortenberg, Germany). A standard amount of protein was mixed with loading dye (cat. no. R1151; Thermo Scientific™, Waltham, MA, USA) and heated for 5 min at 95 °C. This mixture was subjected to sodium dodecyl sulfate polyacrylamide gel electrophoresis and then transferred to a nitrocellulose membrane (Bio-Rad Laboratories, Benicia, CA, USA). After blocking with 5% skimmed milk for 1 h, the membrane was incubated with a primary antibody, VEGF-A (1:1000; cat. no. ab67214615; Abcam, Cambridge, UK), overnight at 4 °C, followed by incubation with the secondary antibody, i.e., horseradish peroxidase-conjugated goat anti-rabbit IgG antibody (1:3000; cat. no. ab6721; Abcam, Cambridge, UK) for 1 h at room temperature. Protein bands were visualized after extensive washing with enhanced chemiluminescence substrate (cat. no. ab65623; Abcam, Cambridge, UK) using the ChemiDoc Imaging System (ChemiDoc^TM^, Bio-Rad Laboratories Inc., Benicia, CA, USA). To verify the equal loading of the proteins, membranes were stripped, reblocked, and reprobed to detect beta-actin (β-actin; 1:1000; cat. no. ab6721; Abcam, Cambridge, UK). The quantification of bands on membrane was carried out using densitometry analysis through ImageJ software version 1.54 (National Institute of Health, http://rsb.info.nih.gov/ij/ [accessed on 15 January 2023]). The integrated optical density (IOD) of each band was calculated and normalized by β-actin as described [26]. All experiments were conducted in triplicate.

#### 2.6.3. Real-Time Polymerase Chain Reaction (RT-PCR)

The suppression of VEGF-A levels in mRNA was determined by RT-PCR. After 24 h treatment of samples, ARPE-19 cells were washed with PBS. Then, total RNA extraction was performed utilizing the AURUM total RNA Mini Kit with DNase digestion (Bio-Rad, Laboratories Inc., Benicia, CA, USA), following the recommended procedure provided by the manufacturer. First-strand cDNA was generated from 1 µg of total RNA using the iScript cDNA Synthesis Kit (Bio-Rad, Laboratories Inc., Benicia, CA, USA). Quantitative real-time PCR (qPCR) was conducted using SYBR Green on the iQ5 Multicolor Real-time PCR Detection System (Bio-Rad, Laboratories Inc., Benicia, CA, USA). The VEGF primer (PrimePCR™ SYBR^®^ Green Assay: VEGF-A, Human, Bio-Rad, Laboratories Inc., Benicia, CA, USA) was used as the target gene, while a mixture of forward primer 18sRNA-5584 (GTAACCCGTTGAACCCCATT) and reverse primer 18sRNA-5734 (CCATCCAATCGGTAGTAGCG) was used for reference genes. The final reaction mixture consisted of 1 µL of each primer, 0.5 µL of cDNA, and 5 µL of iTaq Universal SYBR Green Supermix (Bio-Rad, Laboratories Inc., Benicia, CA, USA), with the remaining mixture volume adjusted to 10 µL using RNase-free water. All reactions were conducted in triplicate. RT-PCR was carried out in a thermal cycler (CFX96^TM^ Real-Time System, Bio-Rad, Laboratories Inc., Benicia, CA, USA) with an initial 3 min hot start denaturation step at 95 °C, followed by 40 cycles at 95 °C for 2 s and at 60 °C for 20 s. Throughout the reaction, fluorescence, and consequently the quantity of PCR products, were continuously monitored via CFX Maestro^TM^ software version 2.3 (Bio-Rad, Laboratories Inc., Benicia, CA, USA). The samples were compared using the relative cycle threshold (Ct) method. After normalization, the levels of increase or decrease were determined with respect to controls, using the formula 2^−ΔΔCt^, where ΔCt is (gene of interest Ct) − (reference gene Ct), and ΔΔ Ct is (ΔCt experimental) − (ΔCt control) [26,27].

### 2.7. Ex Vivo Porcine Transscleral Permeation of CCB

The ex vivo ocular permeation study was conducted using vertical Franz diffusion cells (NK laboratory, Bangkok, Thailand) across the porcine sclera. The receptor phase consisted of 2.0% (*w*/*v*) γCD in PBS (pH 7.4). γCD was added to the receptor phase to maintain a sink condition. A volume of 1.5 mL of CCB/γCD MS was placed in the donor compartment, and the system was maintained at 35 ± 0.5 °C with continuous agitation at 750 rpm throughout the test. CCB in PBS suspension served as a reference formulation. At specific time points, i.e., 1, 2, and 4 h, the sclera was removed and washed three times with ultrapure water and PBS. The amount of CCB retained in each sclera was extracted by cutting them into small pieces and sonicating them in methanol for 30 min. Subsequently, the concentration of CCB was analyzed using the validated HPLC method described in our previous report [16].

### 2.8. Statistical Analysis

All quantitative data were presented as mean ± standard deviation (SD). The statistical analysis was conducted using SPSS version 16.0 software. Initially, the normality of the data distribution was assessed using the Shapiro–Wilk test. The results indicated a normal distribution of the data, justifying the use of a one-way ANOVA for the analysis. Subsequently, we performed a one-way ANOVA followed by Tukey’s post-hoc test. Statistical significance was set at a *p*-value of 0.05.

## 3. Results and Discussion

### 3.1. Physicochemical Characteristics of CCB Eye Drop MS

Table 1 displays the physicochemical characteristics of CCB eye drop MS. These CCB eye drop MS exhibited milky white suspensions. The pH values for both CCB eye drop formulations were determined to be 7.40 after adjustment with sodium hydroxide, which is well tolerated by the eye [28]. The tonicity of the formulations was adjusted by NaCl to be isotonic (260–330 mOsm/kg). The viscosities of these CCB ophthalmic preparations are close to the upper limit of the optimal range, which falls between 15 and 25 cps [29]. This maximum viscosity is able to enhance drug contact time with the ocular surface and keeps the particles well suspended. The surface tension of CCB/RMβCD MS fell within the normal physiological range of lacrimal fluid’s surface tension (40–46 mN/m) [30], whereas CCB/γCD MS exhibited slightly higher surface tension than the physiological range. Surface tension exceeding the physiological range is associated with dry eyes and can lead to tear film instability [31]. Both of the developed CCB eye drop MS were easily redispersed, ensuring the uniform dispersion of solid drug particles in the aqueous vehicle.

It is worth noting that the fraction of CCB dissolved in the aqueous CCB eye drop MS containing RMβCD was significantly higher compared to that containing γCD (approximately 32 times higher). On the other hand, in the formulation containing RMβCD 49% of the drug was in the solid fraction (free CCB and solid CCB/CD complex), whereas this figure was 98% in the formulation containing γCD. All of these physicochemical parameters aligned with previous studies and met the acceptance criteria.

### 3.2. In Vitro Hemolytic Activity

**Figure 1:**
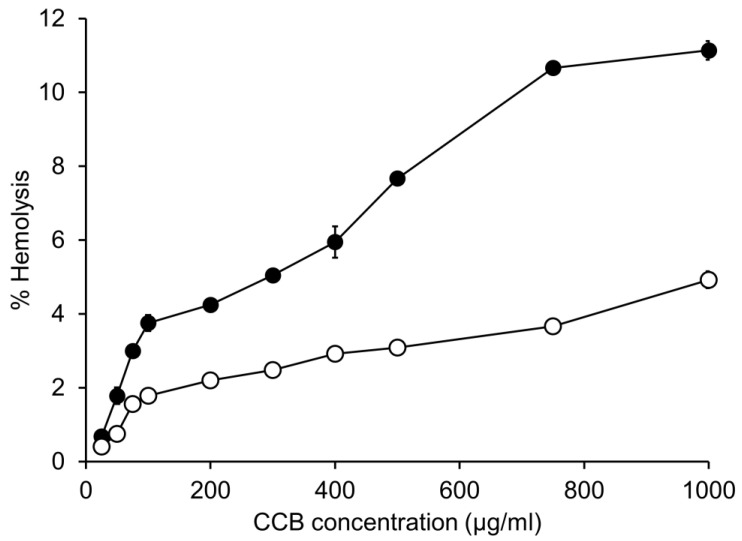
In vitro hemolytic study of sheep red blood cells at the various concentrations of CCB in eye drop MS: CCB/RMβCD MS (●), CCB/γCD MS (○). Data are presented as mean ± S.D., *n* = 3. Note that the error bars are smaller than the symbols’ sizes.

The % hemolysis of CCB/RMβCD and CCB/γCD MS is depicted in Figure 1 and can be regarded in a dose-dependent manner. A higher % hemolysis was observed in CCB/RMβCD eye drop MS compared to that in CCB/γCD eye drop MS. It was observed that the hemolysis of CCB/RMβCD MS (11.1 ± 0.3%) was two times higher than that of CCB/γCD MS (4.9 ± 0.2%) at a CCB concentration of 1000 μg/mL. At this CCB concentration, RMβCD was used at a concentration of 1.5% (*w*/*v*), while γCD was used at a concentration of 2% (*w*/*v*).

Based on previous investigations of in vitro permeation of CCB in eye drop MS through semipermeable and scleral membranes, the permeation flux of CCB from CCB/RMβCD eye drop MS was 29 and 50 times higher than from CCB/γCD eye drop MS, respectively. This difference was attributed to the higher dissolved fraction of CCB in CCB/RMβCD MS, which contributed to drug release regulation and a greater likelihood of binding with the RBC membrane, resulting in increased toxicity. In fact, RMβCD is a lipophilic CD and has a strong affinity to cholesterol. Therefore, it has the ability to extract cholesterol from blood cell membranes, leading to hemolysis even at low concentrations [32].

### 3.3. Hen’s Egg Test-Chorioallantoic Membrane (HET-CAM)

**Figure 2:**
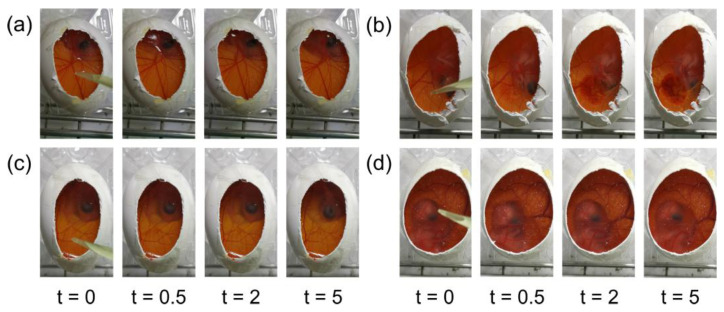
Photographs of HET-CAM at 0, 0.5, 2 and 5 min postinstillation at room temperature: (**a**) 0.9% NaCl (negative control); (**b**) 0.1 M NaOH (positive control); (**c**) CCB/RMβCD MS; and (**d**) CCB/γCD MS.

The HET-CAM test is an alternative test to the Draize test, which is used to evaluate the potential eye irritancy of ocular formulations [33]. The IS values of the CCB/CD eye drop MS were evaluated and compared with those of the positive and negative controls. This HET-CAM experiment can be considered valid because the positive control exhibited an IS value of 17.0 ± 0.0 (Figure 2b), indicating severe irritation, while the negative control showed an IS of 0, demonstrating no irritation (Figure 2a). Applying CCB/RMβCD MS and CCB/γCD MS did not produce any visible signs of irritation or vascular damage, indicating that the developed CCB eye drop MS were safe for ocular drug delivery (Figure 2c,d). Therefore, both CCB eye drop MS were considered suitable for further in vitro cytotoxicity assays.

### 3.4. Short-Time Exposure (STE) Test

The STE test was conducted to assess the cytotoxicity of CCB eye drop MS and to provide eye irritation information similar to that provided by the Draize test conducted on rabbits [19]. Table 2 displays the %CV of SIRC cells after 5 min exposure to 5% and 0.05% CCB eye drop MS, and the scores represent the degree of eye discomfort. The overall score for ocular irritation potential associated with CCB eye drop MS was found to be 1. This indicates that treatment with both CCB eye drop MS and their respective blanks resulted in % CV exceeding 80%. Based on these results, both CCB/CD eye drop MS were classified as mild irritants and considered suitable for ocular drug delivery.

### 3.5. Cell Viability (CV) Test

**Figure 3:**
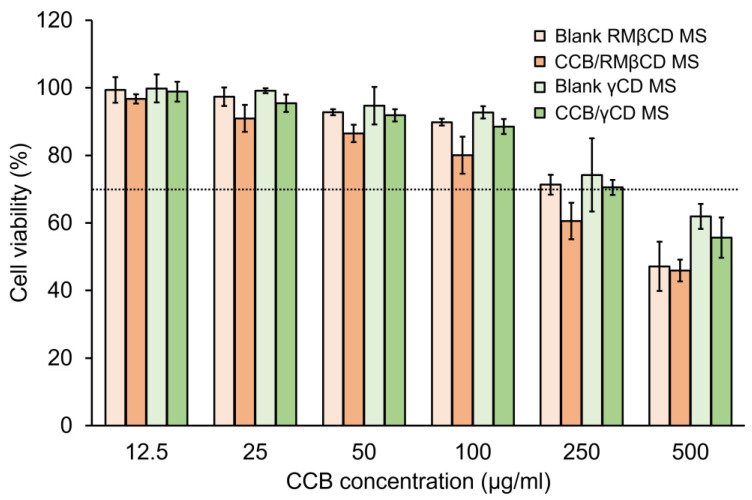
Viability of ARPE-19 cells after 24 h incubation with CCB/RMβCD MS, CCB/γCD MS and their respective blanks. Data are presented as mean ± S.D., *n* = 3.

The in vitro cytotoxicity of CCB/RMβCD MS and CCB/γCD MS on the ARPE-19 cells was further determined using the MTT assay, which was considered cytotoxic when % CV was less than 70% [34,35]. Both CCB/CD eye drop MS and their respective blanks were incubated with ARPE-19 cells at concentrations ranging from 12.5 to 500 µg/mL for 24 h. In all cases, CCB eye drop MS exhibited slightly higher cytotoxicity than their respective blanks. Additionally, the CCB/γCD MS showed lower toxicity compared to CCB/RMβCD MS. CCB/RMβCD MS showed a cytotoxic effect to ARPE-19 cells, with % CV falling below 70% at a CCB concentration of 250 μg/mL, whereas CCB/γCD MS exhibited toxicity only at a CCB concentration of 500 μg/mL (two-fold concentration) (Figure 3). On the other hand, according to the CD concentrations in both drug-free and CCB/CD MS conditions, RMβCD at the concentration of 0.375% (*w*/*v*) and γCD at the concentration of 1% (*w*/*v*) were toxic to ARPE-19 cells. It was confirmed that the lipophilic nature of RMβCD can interact with the cell membrane and induce cell death, consistent with the in vitro hemolysis assay results. From these resulting data, CCB eye drop MS at 100 μg/mL was selected as a safe concentration for further drug efficacy studies.

### 3.6. Western Blotting Assay and Real-Time Polymerase Chain Reaction (RT-PCR)

**Figure 4:**
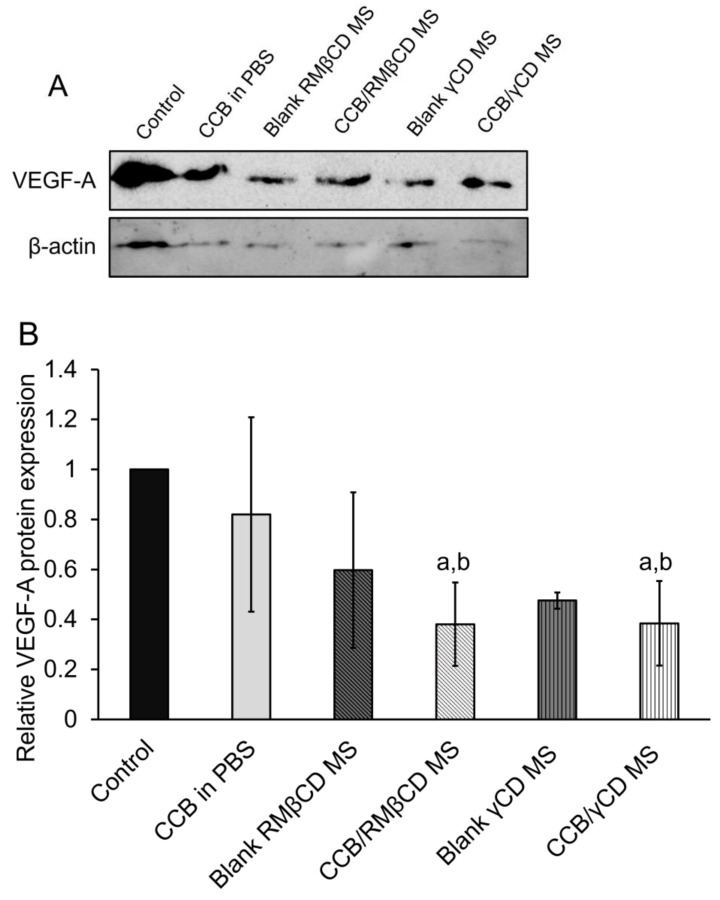
Western blot analysis of VEGF-A protein levels (**A**). β-actin was used as a loading control. Densitometry analysis of VEGF-A protein levels at 100 μg/mL CCB (**B**). Data are presented as mean ± S.D., *n* = 3. ^a^: *p* < 0.05 compared with the values of hypoxic control group and ^b^: *p* < 0.05 compared with the values of CCB in PBS group.

The ARPE-19 cells were subjected to hypoxia with CoCl_2_ for 24 h and then incubated with CCB/CD eye drop MS, their respective blanks, or CCB in PBS. The protein levels and VEGF-A mRNA were examined in the cells using western blot and RT-PCR assays. Pierce et al. (1995) [36] demonstrated that VEGF-A mRNA levels, followed by VEGF protein, increase in a mouse model with relative retinal hypoxia. In Figure 4A, the variation in β-actin band intensity is likely due to the membrane stripping process, which was carried out after VEGF protein detection and subsequent re-probing of the membrane for β-actin, potentially resulting in some protein loss and variable band intensities. However, each sample contained an equal total protein content (100 µg/well) loaded into the wells before running gel electrophoresis, and the quantitative protein expression calculations were determined based on relative protein intensities normalized to the control. In Figure 4B, we found that CCB suspended in PBS had a slight effect on VEGF-A levels, possibly due to the low aqueous solubility of CCB. Surprisingly, drug-free CD-based MS exhibited a lowering effect on VEGF-A protein levels after treatment. In these drug-free CD-based MS, the concentration of γCD was 0.2% (*w*/*v*), while RMβCD was present at 0.15% (*w*/*v*). Therefore, we speculate that the excipients in eye drop formulations partially suppress hypoxia-induced VEGF expression in ARPE-19 cells. There is a report demonstrating that the injection of sodium hyaluronate inhibited the expression of vascular endothelial growth factor receptor-2 (VEGFR-2), but it did not have any impact on reducing the expression of VEGF mRNA in cartilage [37]. Additionally, the incorporation of CCB in these CD-based eye drop formulations significantly suppressed VEGF-A expression (*p* < 0.05). Sun et al. (2017) [38] investigated the signaling mechanism involved in regulating the hypoxia-induced expression of HIF-1α and VEGF through the PI3K/AKT pathway in RPE cells and found that CCB treatment suppressed the activation of this pathway and diminished the protein levels of HIF-1α and VEGF.

**Figure 5:**
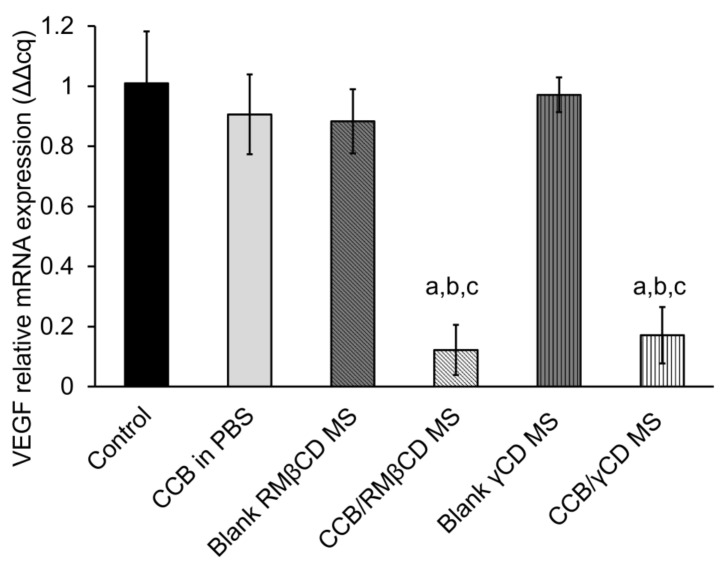
Expression of VEGF-A mRNA in hypoxic ARPE-19 cells. VEGF mRNA levels were determined by RT-PCR at 100 μg/mL CCB. Data are presented as mean ± SD, *n* = 3. ^a^: *p* < 0.05 compared with the values of hypoxic control group, ^b^: *p* < 0.05 compared with the values of CCB in PBS group, and ^c^: *p* < 0.05 compared with their respective blank MS group.

The VEGF-A mRNA levels after treatment with CCB eye drop MS were observed by RT-PCR. In comparison to CCB suspended in PBS, significant suppression of VEGF-A mRNA levels in both CCB/CD eye drop MS (*p* < 0.05, Figure 5) was observed. This reduction can be attributed to the high CCB loading and the CCB/CD nanoaggregates, which provide better intracellular uptake than free CCB in PBS. There was an insignificant difference in anti-VEGF efficacy between these two CCB/CD eye drop MS (*p* > 0.05). Therefore, CCB/CD eye drop MS has the potential to deliver CCB to the retina for AMD and DR treatment. Based on the results of the cytotoxicity studies, CCB/γCD MS exhibited less hemolytic activity and lower cytotoxicity to the ARPE-19 cells compared with CCB/RMβCD MS, and thus, it was selected for further investigation.

### 3.7. Ex Vivo Transscleral Diffusion Studies

**Figure 6:**
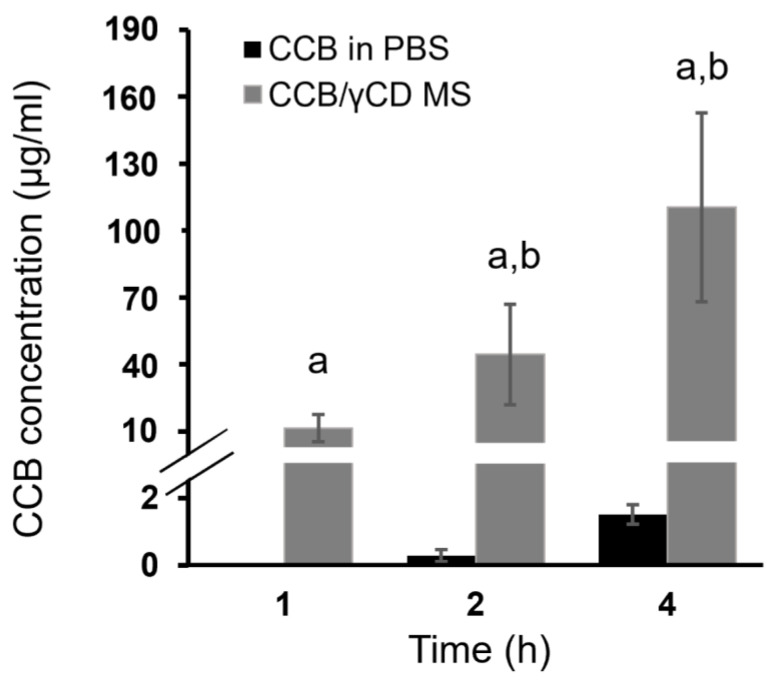
Ex vivo transscleral diffusion of CCB suspended in PBS and CCB from CCB/γCD MS through the porcine sclera. Data are presented as mean ± SD, *n* = 3–4; ^a^: *p* < 0.05 compared with the values of CCB in PBS, and ^b^: *p* < 0.05 compared with the values in different time intervals.

Figure 6 displays the results of ex vivo transscleral diffusion of CCB (CCB in PBS or CCB/γCD MS) through the porcine sclera. After 1 h, the amount of CCB diffused from CCB/γCD MS into sclera tissue was found to be 11.31 ± 4.11 μg/mL, while in the case of CCB in PBS, it was undetected. The diffusion of CCB was observed to increase over time in the cases of both CCB/γCD MS and CCB in PBS. After 4 h, CCB/γCD MS showed a significantly higher amount of CCB retained in sclera tissue (112.47 ± 37.27 μg/mL) compared to CCB in PBS (1.51 ± 0.29 μg/mL) (*p* < 0.05). This improvement (approximately 74 times higher than CCB in PBS) might be attributed to (1) increasing the aqueous solubility of CCB through the CCB/γCD inclusion complex; (2) the addition of HA, a mucoadhesive polymer, which adheres to the sclera membrane surface and provides sustained CCB release; and (3) increasing the contact area through the formation of nano and small microparticles (i.e., increasing the surface area via particle size reduction), resulting in an increased quantity of CCB deposited in the deeper scleral tissues.

## 4. Conclusions

This study confirmed that our developed CCB/CD eye drop MS can be reproducible for manufacturing with reliable physicochemical characteristics. Both CCB/RMβCD MS and CCB/γCD MS are well tolerated by the eyes, with low hemolytic activity, cytocompatibility with corneal and retinal cell lines, and no signs of irritation. Due to the favorable toxicological profiles of γCD, CCB in γCD-based MS exhibited lower toxicity than the eye drops containing RMβCD. Regarding our CD-based technology, the diffusion of CCB into scleral tissue was successfully achieved. Our CCB/CD eye drop MS demonstrates that CCB has anti-VEGF efficacy, effectively reducing VEGF protein and mRNA levels in hypoxic RPE cells. Based on these findings, it is reasonable to propose that our developed eye drop MS containing ternary CCB/CD/HA complexes has promising properties for the potential treatment of AMD and DR, and this warrants further investigation through in vivo studies.

## Figures and Tables

**Table 1:** Physicochemical parameters of CCB eye drop MS.

Parameters	CCB/RMβCD MS	CCB/γCD MS
Appearance	Milky white suspension	Milky white suspension
pH	7.40 ± 0.06	7.39 ± 0.02
Osmolality (mOsmol/kg)	299.67 ± 2.08	301.00 ± 2.00
Viscosity (cPs) ^a^	22.76 ± 1.53	27.53 ± 1.72
Surface tension (mN/m) ^a^	45.32 ± 0.15	48.35 ± 2.07
Redispersion time (s)	28.00 ± 2.65	20.67 ± 1.15
Total drug content (%)	102.51 ± 0.07	96.34 ± 0.78
Dissolved CCB content (%)	51.44 ± 4.11	1.61 ± 0.12

^a^ determined at 25 °C.

**Table 2:** Scores obtained from the STE test of CCB eye drop MS (mean ± S.D., *n* = 4).

Concentrations of Test Samples	Test Samples	%CV of SIRC Cells	Criteria for Scoring	Obtained Scores
(I) 5%	Blank RMβCD MS	92.95 ± 1.70	If CV > 70%: scored = 0 If CV ≤ 70%: scored = 1	0
	Blank γCD MS	94.51 ± 2.76		0
	CCB/RMβCD MS	77.96 ± 4.57		0
	CCB/γCD MS	84.88 ± 3.15		0
(II) 0.05%	Blank RMβCD MS	99.30 ± 1.29	If CV > 70%: scored = 1 If CV ≤ 70%: scored = 2	1
	Blank γCD MS	99.68 ± 1.00		1
	CCB/RMβCD MS	89.74 ± 2.21		1
	CCB/γCD MS	91.22 ± 1.41		1
Total score (I and II)				
Blank RMβCD MS				1
Blank γCD MS				1
CCB/RMβCD MS				1
CCB/γCD MS				1

## Data Availability

Data are contained within the article.
